# Systematic review and meta-analysis of cancer risks in relation to environmental waste incinerator emissions: a meta-analysis of case-control and cohort studies

**DOI:** 10.4178/epih.e2022070

**Published:** 2022-09-01

**Authors:** Kiook Baek, Jong-Tae Park, Kyeongmin Kwak

**Affiliations:** 1 Department of Occupational and Environmental Medicine, Yeungnam University Hospital, Daegu, Korea; 2 Department of Occupational and Environmental Medicine, Korea University Ansan Hospital, Ansan, Korea

**Keywords:** Dioxin, Incineration, Neoplasms, Meta-analysis

## Abstract

**OBJECTIVES:**

Various toxic substances can be generated from incinerators, exposing nearby residents, and epidemiological studies have shown wide variations in risk estimates for cancer risk in populations living close to incinerators.

**METHODS:**

Following the Preferred Reporting Items for Systematic Reviews and Meta-Analyses (PRISMA) guidelines, a literature search and systematic review were conducted to identify studies conducted on general populations exposed to environmental incinerator emissions and cancer outcomes. Meta-analysis was performed according to the cancer types for which 2 or more studies were reported. Subgroup analysis was done for sex, the exposure estimation method, the study period, and the type of outcome.

**RESULTS:**

Eleven studies were found for the qualitative review and meta-analysis. Seven studies had a case-control design, and 4 had a cohort design. The pooled effect size was not significant for breast, colorectal, liver, lung, lymphohematopoietic, stomach, bladder, central nervous system, and laryngeal cancers, non-Hodgkin lymphoma, sarcoma, leukemia, and all cancers. In the subgroup analysis, the pooled effect size of laryngeal cancer in females was 1.82 (95% confidence interval, 1.10 to 3.01), although only 2 studies were identified.

**CONCLUSIONS:**

The meta-analysis did not provide evidence of an increased risk for any cancer among populations living near waste incinerators, except for laryngeal cancer in females. However, since relatively few studies were reviewed and some cancer types showed significant increases in individual studies, this evidence needs to be updated regularly.

## INTRODUCTION

Populations living near incinerators may be exposed to various pollutants (e.g., ash, odor, dust, or spores) [1], including carcinogens such as dioxins and heavy metals [2,3]. Although regulations on incinerators have been strengthened and pollutant emissions and exposures are decreasing [4,5], concerns regarding the potential health effects of living near incinerators persist. To date, no clear consensus exists on the increase in cancer risk owing to long-term exposure to low concentrations of carcinogens [6]. Debate continues regarding the extrapolation and application of the estimated carcinogenic effect at high concentrations to the risk of cancer in settings of low-dose exposure [7]. It is challenging to confirm the level or even the existence of the carcinogenic threshold at low concentrations of toxicants, and the estimation of carcinogenic risk through extrapolation is also uncertain [8].

Many studies, including well-designed ones, have reported that living near incinerators poses a cancer risk; however, some studies with relatively unsophisticated designs have also shown a low evidence level [9]. It is impossible and inefficient for the public and decision-makers to review all the literature and data accumulated to date. In addition, there is a risk of selectively obtaining and citing research results that support different claims. Thus, studies with a high level of evidence and integrated results are warranted.

Despite a large number of systematic reviews on the risk of environmental exposure to toxicants from incinerators [10-12], no meta-analysis has been reported. Therefore, we performed a systematic review and meta-analysis of case-control and cohort studies among populations living near incinerators, focusing on cancer risk.

## MATERIALS AND METHODS

### Study design, protocol, and registration

This systematic review and meta-analysis was performed according to the Preferred Reporting Items for Systematic Reviews and Meta-Analyses (PRISMA) guidelines [13,14]. The protocol was registered in the International Prospective Register of Systematic Reviews on January 5, 2022 (CRD42022292049) [15].

### Literature search

PubMed, Embase, and Web of Science were searched for articles published up to December 3, 2021. The search strategy for each database used during the literature search is shown in Table 1. Those with duplicate data were excluded; the title and abstract and then the full-text were checked in accordance with the inclusion and exclusion criteria described below. References from the included literature and the literature cited in the included literature were manually checked to retrieve reports from the initial search using the same method that was used for assessing the initial search results. Individual studies and the corresponding extracted data were reviewed independently by 2 unblinded authors, KB and KK. If there were different opinions on the inclusion or exclusion of the papers, a vote was taken by all 3 authors.

### Inclusion criteria

We included studies on the increase in human cancer incidence among general populations living near incinerators or those exposed to emissions from incinerators, which provided risk estimates and confidence intervals (CIs) for comparisons between non-dose or low-dose exposure groups. We included studies reporting any human cancer risk outcome. Studies with all types of incinerators were searched, regardless of the construction period, generation, or incineration material. Only case-control and cohort studies were included.

### Exclusion criteria

The following studies were excluded: ecological studies; animal studies; studies on occupational exposure to incinerator emissions; studies on risk assessments or exposure assessments without cancer-related data; review articles (including systematic reviews); non-original research (such as case reports, case series, commentaries, and conference abstracts); and studies on general industrial pollution wherein incinerator emissions were not evaluated.

### Data extraction

From the included studies, data regarding the name of the first author, publication year, sex of the participants, study design, region, size, period of the study, outcomes, exposure assessment method, effect size, and 95% CIs were extracted. If an exposure was classified into several exposure levels, the effect size of the lowest compared with that of the highest estimated exposure group was extracted. When the exposure was classified based on the distance from the incinerator, the effect size of the nearest area compared with that of the farthest from the incinerator was extracted. If the effect size and CI were not provided explicitly in a study, they were calculated using data presented in the article. Cases in which several effect sizes were stratified by sex or age were pooled. Among studies reporting both adjusted and unadjusted values for confounders, the adjusted values were extracted.

### Quality assessment

We evaluated each article using the Newcastle–Ottawa Scale (NOS). The NOS contains 8-item categories in three components: selection, comparability, and outcome (for cohort studies) or exposure (for case-control studies) [16]. It is scored on a 0-point to 9-point scale, with 7-9 points indicating high quality, 4-6 points indicating intermediate quality, and 0-3 points indicating low quality [17]. Individual studies were independently assessed by 2 authors: KK and KB. If there were different opinions on whether to include or exclude a study, a vote was taken by all authors.

### Statistical analysis

The pooled effect size and the corresponding 95% CIs were calculated for each cancer type reported in at least 2 studies. The effect sizes for all cancer types were pooled. Although the outcomes of the studies that included various cancers were heterogeneous, this method has been previously used in the literature [18,19]. If duplicate effect sizes were reported, the effect size for the upper category was extracted and analyzed. Although the effect sizes varied in terms of whether they were reported as the hazard ratio (HR), rate ratio (RR), or odds ratio (OR), the results were pooled together in consideration of the rare disease assumption [20]. RRs and HRs for incidence or hospitalization from cohort studies were pooled with ORs from case-control studies. A random-effects model was used considering the heterogeneity of environmental epidemiological studies [21]. The I2 statistics and p-values of the Cochran Q test for each analysis are presented. Funnel plots and the Egger test were used to evaluate publication bias. Meta-analyses were conducted using Stata version 15 (StataCorp., College Station, TX, USA) with the “metan” command. Publication bias was assessed using R version 3.6.3 (https://R-project.org) with the “metafor” package.

### Subgroup analysis

The following subgroup analyses were performed: (1) A meta-analysis was done with stratification by sex for studies that reported the effect size by sex. (2) Since the method of exposure estimation was different for each study, cases where the exposure was evaluated by modeling emissions components were analyzed separately. (3) A meta-analysis was performed of studies reporting the effect size for mortality. (4) A meta-analysis was performed according to the time of the start of exposure (before and after 2000), considering the lower pollution emissions of recently built incinerators [11].

### Ethics statement

No ethical approval is required since this study was based on published articles and did not involve human subjects.

## RESULTS

### Characteristics of the included studies

**Figure 1.**
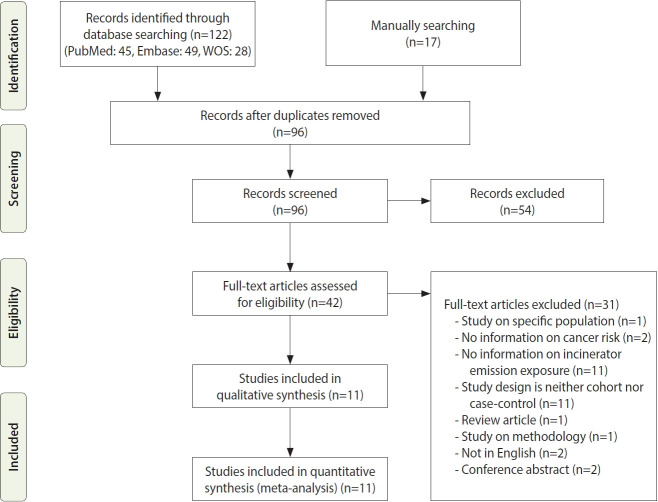
Preferred Reporting Items for Systematic Re views and Meta-Analysis (PRISMA) flaw diagram of the selection of studies for the systematic review. WOS, Web of Science.

With search terms, 122 studies were identified from databases. Sixteen studies were manually searched from citations and references. Duplicate publications were removed, and the remaining articles were screened. We excluded 54 articles after screening their abstracts and reviewed the full-text of 42 articles. Articles for which we performed full-text review are presented in Supplementary Material 1. Finally, 11 articles were included in the final literature review and meta-analysis (Figure 1).

Briefly, 7 were case-control studies, and 4 were cohort studies. The study periods spanned from 1979 to 2015. Among the cohort studies, 1 study reported only the incidence; 2 studies reported mortality and hospitalization; and 1 study reported mortality, incidence, and hospitalization as outcomes. ORs were extracted from the case-control studies. As for the types of incineration facilities, exposure evaluation was performed for municipal waste in 6 studies, industrial waste in 2, and medical, municipal, sewage, and hazardous waste in 1. There was no mention of the type of incinerator in 1 study. One study evaluated incinerators and industrial exposure without distinction [22]; however, the incinerator accounted for a large proportion of exposure, and the exposure assessment (performed using dispersion modeling) was of high quality; thus, we included this study. One case-control study reported the modeling of the-estimated relative risk according to the distance from the incinerator in cases based on lung cancer autopsy results [4]. Other detailed information on each study is presented in Table 2. The NOS evaluation results are provided as online Supplementary Material 2.

### Effect size extraction and meta-analysis

ORs from case-control studies and RRs/HRs from cohort studies were extracted and pooled. Pronk et al. [23] reported the effect sizes of 4 incinerators (medical, municipal, sewage, and hazardous waste) and other industrial facilities. Because the exposed populations of the 4 incinerators overlapped, the effect size (OR) for medical waste incinerators, which caused the most exposure, was extracted.

**Figure 2.**
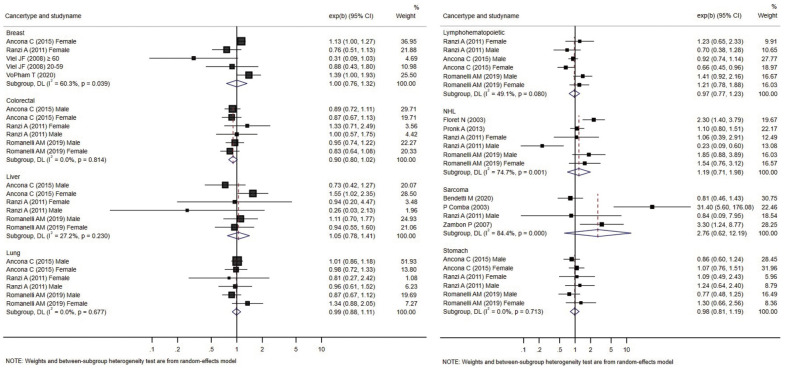
Forest plot of studies included in the meta-analysis of living close to an incinerator and the risk of cancer reported in at least 2 studies by cancer type. exp(b), indicates effect size of each study; CI, confidence interval.

**Figure 3.**
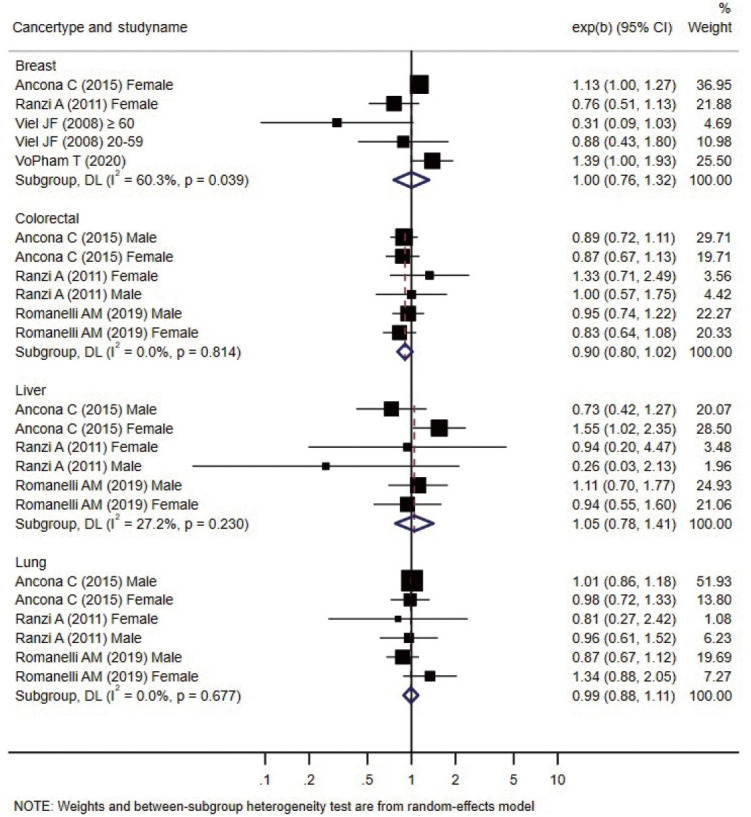
Forest plot of studies included in the meta-analysis of living close to an incinerator and the risk of cancer reported in 2 studies by cancer type. exp(b), indicates effect size of each study; CI, confidence interval.

Forest plots presenting individual and pooled effect sizes for breast, colorectal, liver, lung, lymphohematopoietic, and stomach cancers, as well as non-Hodgkin lymphoma (NHL) and soft tissue sarcoma, are presented in Figure 2. Forest plots for the outcomes reported in 2 studies (bladder, central nervous system [CNS], laryngeal cancers, leukemia, and all cancers) are presented in Figure 3. The meta-analytic pooled effect sizes for all cancer types combined are shown in Supplementary Material 3. A funnel plot and coefficient from the Egger test are presented in Supplementary Material 4.

### Summary by cancer type

#### Breast cancer

The risk of breast cancer was reported in 4 studies, all performed in females. VoPham et al. [24] reported that the incidence of breast cancer was significantly higher in individuals living near incinerators among a prospective cohort of nurses. For individuals living within 3 km, 5 km, and 10 km of municipal solid waste incinerators, the HRs were 1.20 (95% CI, 0.86 to 1.68), 1.25 (95% CI, 1.04 to 1.52), and 1.15 (95% CI, 1.03 to 1.28), respectively. The HRs tended to increase as the period of residence near the incinerator increased. Compared with the group without a period of residence at a distance of 3 km near municipal solid waste incinerators, the HR increased by 1.07 (95% CI, 0.77 to 1.49) for 1-6 years and 1.39 (95% CI, 1.00 to 1.93) for those with more than 6 years of residence. In a case-control study conducted by Viel et al. [25], the results were stratified between the 20-year to 59-year and ≥ 60-year age groups, and no significant relationship with residence near incinerators was found in either group. Two retrospective cohort studies did not confirm a significant relationship [26,27]. The pooled effect size was not significant.

#### Non-Hodgkin lymphoma

In a case-control study conducted in France by Floret et al. [28], there was a significantly higher incidence of NHL in individuals living near incinerators. In another case-control study from the United States no significant difference was identified in a case-control study reported later [23]. Two retrospective cohort studies reported risk estimates for NHL. Romanelli et al. [29] reported no significant increase in the HRs, while Ranzi et al. [27] reported that the risk of NHL was significantly lower in males living near incinerators. The pooled effect size was not significant.

#### Soft tissue sarcoma

In a case-control study conducted by Comba et al. [30], the OR for soft tissue sarcoma was calculated according to the distance from an incinerator, and the result obtained was significant (OR, 31.4; 95% CI, 5.6 to 176.1) for the group living within 2 km of an incinerator compared to the group living more than 5 km away. There were 5 cases and 1 control in the group residing within 2 km of an incinerator and 7 cases and 44 controls in the group residing more than 5 km away.

In 2007, Zambon et al. [22] conducted a case-control study in Italy and calculated the OR considering the estimated dioxin exposure level and period through dispersion modeling. The study included 172 patients and 405 controls. The OR (3.30; 95% CI, 1.24 to 8.77) was significantly higher in the group exposed to ≥ 6 fg/m^3^ of polychlorinated dibenzo-p-dioxins and dibenzofurans for more than 32 years than in the group exposed to < 4 fg/m^3^ of polychlorinated dibenzo-p-dioxins and dibenzofurans for less than 32 years. Ten cases and 44 controls were exposed to < 4 fg/m^3^ of dioxin for less than 32 years, while 20 cases and 26 controls in the control group were exposed to ≥ 6 fg/m^3^ of dioxin for ≥ 6 years. In the assessment that did not consider the exposure period, a significant result (OR, 2.41; 95% CI, 1.04 to 5.59) was reported in the female group with exposure to ≥ 6 fg/m^3^ of dioxin compared to that in the group with exposure to < 4 fg/m^3^ of dioxin. However, in the study by Benedetti et al. [31], residential history was classified in various manners, but no significant increase was observed with any method. The OR calculated in the exposure group for the population exposed after 1961, excluding 10 years before the diagnosis, was 0.81 (95% CI, 0.46 to 1.43); when the time-window and high exposure period (1961-1991) ere considered in the exposure history, the analysis showed no significant decrease in risk (OR, 0.57; 95% CI, 0.27 to 1.21). The pooled effect size was not significant.

#### Colorectal, stomach, lymphohematopoietic, and liver cancers

Colorectal, stomach, lymphohematopoietic, and liver cancers were reported in 3 retrospective cohort studies [26,27,29] that analyzed various cancers; no significant increase in the incidence or mortality was observed in individual studies. All 3 studies were conducted in Italy. Ancona et al. [26] reported the outcomes in Rome based on data from the regional hospital information system and the regional registry of causes of death. Ranzi et al. [27] used data from the cancer database, hospital admissions database, and regional mortality database in Forlì. Romanelli et al. [29] reported cancer outcomes based on the regional hospital information system and regional mortality registry data in Pisa. In all 3 studies, codes from the International Classification of Diseases (ICD), ninth revision, were used to analyze the cancer type as a health outcome. The pooled effect sizes were not significant.

#### Lung cancer

Three retrospective cohort studies [26,27,29] reported non-significant changes in lung cancer-specific incidence or mortality. The pooled effect size was not significant. There was no significant increase in the pooled effect size in the meta-analysis.

Biggeri et al. [4] conducted a case-control study that analyzed the change in relative risk according to the distance from the city center, an iron foundry, a shipyard, and an incinerator, with modeling conducted through a point source analysis. Even after adjustments for individual risk factors, the maximum OR (5.9, p< 0.001) was estimated at the point of the incinerator and decreased sharply with distance. However, because this value was not the actual outcome of research and the case definition was death (autopsy), we excluded this study from the meta-analysis presented in Figure 2. This study was pooled in the subgroup meta-analysis for mortality.

#### Laryngeal cancer, bladder cancer, central nervous system cancer, and leukemia

Two studies reported the effect size of living near incinerators on laryngeal cancer incidence. A significant increase in laryngeal cancer-specific mortality and hospital admissions was reported among females in a retrospective cohort study conducted by Ancona et al. [26]. Significant differences in mortality (OR, 1.92; 95% CI, 1.16 to 3.19) and hospital admissions (OR, 1.83, 95% CI, 1.09 to 3.06) were reported among the females exposed to waste incinerator emissions in the cohort. Exposure estimation was modeled using particulate matter less than 10 μm (PM10). The OR was calculated as the difference between the 95th and 5th percentiles. No significant elevation in mortality or hospital admissions was observed among males. Ranzi et al. [27] reported a non-significant association between laryngeal cancer risk and living close to incinerators. Exposure estimation was performed using dispersion modeling for heavy metal exposure. RRs were calculated by stratifying the estimated exposure by quartiles. The number of cases identified as laryngeal cancer was 18 in the first quartile of exposure and 1 in the fourth quartile of exposure. Among males, the RR was 0.15 (95% CI, 0.02 to 1.13). The incidence of laryngeal cancer was 2 in the first quartile of exposure and 1 in the fourth quartile of exposure; and the RR was 1.60 (95% CI, 0.15 to 17.35). In the same study, the RR for mortality could not be calculated because there were no deaths in the first and fourth quartiles among females, while 0 deaths and 6 deaths were observed in the fourth and first quartiles among males, respectively.

Two retrospective cohort studies [26,27] analyzed the risk of bladder cancer in relation to living near incinerators and found no significant results. The results were shown for males and females separately.

CNS cancer was analyzed in 2 retrospective cohort studies [27,29]. In a study with dispersion modeling for heavy metal exposure [27], the risk excess was not significant (RR, 1.35; 95% CI, 0.34 to 5.39) among males comparing the groups with the lowest and highest exposures. No cases were identified in the highest exposure group among females. Romanelli et al. [29] reported a non-significant increase in the incidence of CNS cancer in males (HR, 1.87; 95% CI, 0.54 to 6.44) and a non-significant reduction in CNS cancer incidence in females (HR, 0.38; 95% CI, 014 to 1.05).

Some studies reported the risk of leukemia and myeloma separately from that of lymphohematopoietic cancer. Leukemia was reported in 2 studies [27,29], and the risk was presented separately for males and females; however, no significant difference was found in effect size.

#### All cancers

Studies reporting the risk for all cancers in the original text were analyzed. Two studies calculated the effect size by integrating all types of cancers. Two retrospective cohort studies [26,27] reported the effect size of all cancers based on ICD codes among cohort participants. One study used ICD codes 140-239 to define all cancers [27], while another study used ICD codes 140-208 [26]. The effect size was presented separately by sex. In the meta-analysis, the 95% CI for the pooled effect size overlapped with the null hypothesis.

Although the heterogeneity of the outcome was severe, in the meta-analysis process, the effect size of incidence was pooled regardless of the type of cancer. The pooled effect size was 1.00 (95% CI, 0.94 to 1.06), which was not statistically significant.

### Subgroup analysis

The results of the subgroup analysis are presented in Supplementary Material 5. In the meta-analysis that analyzed only the mortality outcome, a significant increase or decrease in the effect size was not observed. No significant increase or decrease in the effect size was observed in the meta-analysis of studies that estimated exposure through modeling. When stratified based on the study period (time of enrollment), the effect size for cancer did not show a statistically significant result.

In the subgroup analysis by sex, the risk of laryngeal cancer was significantly increased in females (effect size, 1.82; 95% CI, 1.10 to 3.01) with pooled results from 2 studies. Except for laryngeal cancer in females, there were no statistically significant results in the analysis stratified by sex.

## DISCUSSION

The articles reviewed in this study were case-control and cohort studies, which are regarded to have relatively high epidemiological evidence levels among observational study designs. However, the results were inconsistent and varied, which could cause confusion among the public and experts; thus, the need for quantitative pooling of studies has been raised. We conducted a systematic review and meta-analysis of 11 studies presenting the risk of cancer in general populations living near incinerators. There have been reports of clusters of various cancer types related to residence near a waste management site [32], and research continues to be conducted on the increase in cancer incidence in populations residing near incinerators. Individual studies have reported increases in the risk of sarcoma [30], NHL [28], lung cancer [4], laryngeal cancer, and pancreatic cancer [26]. No significant results were noted in a meta-analysis of cancers identified in 2 or more studies. In the subgroup analysis, the pooled risk of laryngeal cancer in females reported in two studies showed statistically significant results, though only 2 studies presented data on laryngeal cancer risk in females. Associations of laryngeal cancer with dioxins [33], PM10 [34], and heavy metals [35] have been reported. Although epidemiologically, laryngeal cancer is a rare disease, it has been suggested to be related to residence near an incinerator since 1990; however, the evidence for this has not been sufficient [36]. There have also been inconsistencies in the evidence gathered since then. Several studies that were not included in this systematic review process due to their ecological study design have reported changes in the risk of laryngeal cancer with residence near an incinerator. One study showed that distance from a plant with an incinerator was associated with the standardized mortality ratio for laryngeal cancer, with an increasing pattern as the distance decreased from the plant area (p= 0.03). However, this result became insignificant after adjusting for socioeconomic status (p= 0.06) [37]. Other ecological-design studies reported no significant change [38] or decrease [39] in the association between the risk of laryngeal cancer and living near an incinerator. The quality of evidence and the direction of effects reported in various studies vary; thus, there is a risk of selective selection in research and the media. Although our study did not draw a clear conclusion, it will help in decision-making and set directions for future research by presenting results with a relatively high evidence level and suggesting a pooled effect. The effect of exposure caused by incinerator emissions on laryngeal cancer requires further investigation, and the accumulation of evidence should be continuously monitored.

The substances emitted by incinerators include PM_10_, dioxins, heavy metals, and nitric oxide (NO) and nitrogen dioxide (NO_2_). Although there are some critical views on the carcinogenic effects of dioxin-like compounds [7,8], it is widely accepted that they pose a cancer risk, according to studies on high-dose exposure groups, such as workers or residents exposed to the Seveso accident [40-42]. The International Agency for Research on Cancer reported that there was sufficient evidence for the carcinogenic effect of 2, 3, 7, 8-tetrachlorodibenzo-para-dioxin for all cancers combined and limited evidence with respect to lung cancer, soft tissue sarcoma, and NHL among individual cancers in humans [43,44]. Studies have sporadically reported the association of dioxin exposure with other cancers, such as breast and rectal cancers [45,46]. However, it is difficult to confirm the dose-response curve and threshold for extrapolation to low-dose exposure [47]. Similarly, heavy metals, such as cadmium, arsenic, and chromium, are widely known carcinogens for lung cancer that can be generated in incinerators [48]. Nonetheless, recent studies on environmental exposure to flue gas near incinerators have shown that levels of pollutants are not substantially higher near incinerators than in the general atmosphere [49]. Even if exposure is higher than in a non-exposed area, it is difficult to assess the effect of long-term exposure to low concentrations of carcinogens in the environment. Although some studies have estimated the risk through substance exposure assessment [50], estimating the risk without epidemiological evidence is insufficient, and these uncertainties cause various obstacles to risk perception and risk communication in residences and establishments around incinerators [17,51,52].

In epidemiological studies of environmental factors, exposure assessment for individuals is a challenging process [53] and has been reported heterogeneously in various studies [54]. For instance, Goria et al. [55] recommended using dispersion modeling to estimate exposure rather than the distance from the source because the results may depend on the exposure assessment methodology used in the analysis of the same data. In general, in spatial analysis, carcinogenic contaminants other than those produced by incinerators may coexist, and attempts have been made to use dispersion modeling to overcome this issue [26,29]. Various substances are generated from incinerators, and exposure estimates for carcinogens, such as dioxins, vary depending on incinerator usage and weather conditions; thus, risk assessment is difficult owing to the high level of uncertainty [10]. In the studies included in this review, distance from the source, region, and dispersion modeling were used to assess individual exposure. However, the indicator materials used for dispersion modeling were diverse, such as dioxins, heavy metals, PM_10_, and NOx, and the modeling methods were heterogeneous. No single tool has been optimized for assessing carcinogens or other toxicants around incinerators. The subgroup analysis was performed by pooling only cases where exposure was estimated by modeling, regardless of the modeling method; however, there was no change in the trend of the results. A more detailed subgroup analysis according to the exposure assessment method is needed, but it was impossible to proceed further because the number of studies was insufficient to stratify the method of exposure assessment.

It is difficult to conclude that incinerator emission exposure is not associated with an increase in other cancer risks based on the results of this study alone. In our review, studies from Italy (n= 7), France (n= 2), and the United States (n= 2) were included. The European Union began to regulate incinerator emissions in earnest from the early 1990s [56], and in the United States, the regulation of incinerators was strengthened after 1995 [57]. Moreover, the degree of regulation and incineration materials are different for each country, resulting in a trend of differences in emissions. The studies included in this meta-analysis were conducted in countries with relatively strong regulations; therefore, the exposure levels were expected to be relatively low [58]. Studies with large weights, such as in our meta-analysis, are relatively recent, and timed after strict emission regulations were implemented in Europe and the United States. It is possible that the carcinogenic effect size may have been underestimated due to the relatively low exposure to emissions in the group classified as “high exposure.” It is necessary to update research results for regions with a relatively high exposure level of pollutants caused by emissions, such as underdeveloped or developing countries. In addition, most studies classified exposure groups based on the place of residence, and even if precise modeling is performed, individual exposure cannot be perfectly estimated. Even given these technical difficulties, there are still insufficient studies that have directly measured exposures or estimated exposures using biological exposure markers.

The limitations of this meta-analysis are that there were variations in the design (participants, exposure estimation, comparator, and outcome) among the studies, and the number of included articles was relatively small. Studies involving different periods, countries, types of incinerators, and incinerated materials were pooled. There are differences in the definitions of disease outcomes in the literature; for instance, the prevalence of sarcoma was low and there were a variety of types. In 1 study, lymphatic vessel sarcoma, nerve sheath sarcoma, and alveolar sarcoma were included in the extraction process using the ICD for Oncology-II morphology code [22], whereas in another study, it was not included [30]. Two studies reported “all cancer” risk, using different selection criteria [26,27]. In addition, there were differences in the confirmation of cancer outcomes across studies, as exemplified by the use of medical reviews, cancer registries, questionnaires, or biopsies and autopsies. Nevertheless, it was difficult to perform meta-regression or further stratified subgroup analyses for various factors because few studies have reported the effect size for each cancer. Although a subgroup analysis was performed based on sex, important general characteristics, such as age, country, and race/ethnicity, were not stratified. Another limitation is that a stratified analysis was not performed on the generation of incinerators, although it was attempted to supplement this gap by stratification according to the enrollment dates of the study subjects.

Moreover, there was a risk of bias in each study’s use of spatial data as exposure variables. Due to the nature of spatial analyses linked to addresses, there was a risk of selection and ecological bias in reflecting actual individual exposure [21,59]. Several studies have been conducted on dioxin biomonitoring near incinerators; however, it was difficult to find case-control or cohort studies reporting both individual biomonitoring and cancer risk.

Atmospheric exposure to toxicants is low compared to workplace standards; however, continuing exposure to air pollutants, such as PM10 and dioxin, can cause various subclinical health effects [60,61]; thus, concerns regarding the cancer risk of individuals residing near incinerators persist [62]. Authorities such as the US National Research Council [63] insist that the actual health risk of individuals residing near modern incinerators is minimal to moderate in normal, controlled operating conditions; nonetheless, uncertainty and potential risk remain. To overcome these uncertainties, continuing epidemiological and mechanistic studies, as well as systematic literature reviews of such studies, are required to provide more clear information to the public and policy-makers. It is encouraging that studies with a high evidence level that synthesize various disease data, such as health insurance data and cancer registration data, and atmospheric modeling data are being conducted, and evidence should continue to be collected and updated. The systematic search strategy used herein constitutes a resource for regular literature searchers using the suggested strategy to keep results up to date.

## CONCLUSION

Several systematic reviews on the health risks, including cancer risks, and health effects of living near incinerators have been previously conducted; however, a quantitative synthesis has not yet been performed. So far, there is a lack of evidence of elevated risk of specific cancers after pooling the effect sizes by cancer type, except for laryngeal cancer in females. However, the evidence for each cancer type is relatively small-scale; therefore, it is difficult to conclude that sufficient evidence has been gathered. It is necessary to monitor and update the evidence on a regular basis in the future.

## Figures and Tables

**Table 1.** Systematic review search strategy

	PubMed		Embase		Web of Science (including ESCI)
#1	Incineration[mh] OR incinerat*[tiab] OR thermal destruction*[tiab]	#1	incineration/de OR (incinerat* OR 'thermal destruction*'):ti,ab	#1	TS=(incinerat* OR "thermal destruction*")
#2	Refuse Disposal[mh:noexp] OR refuse disposal[tiab] OR waste disposal[tiab] OR waste plant*[tiab] OR waste treatment[tiab]	#2	('waste disposal'/exp OR ('refuse disposal' OR 'waste disposal' OR 'waste plant*' OR 'waste treatment'):ti,ab)	#2	TS=("refuse disposal" OR "waste disposal" OR "waste plant*" OR "waste treatment")
#3	#1 OR #2	#3	#1 OR #2	#3	#1 OR #2
#4	Occupational Exposure[mh:noexp] OR Occupational Health[mh] OR occupational exposure*[ti] OR worker*[ti]	#4	('occupational health'/exp OR ('occupational exposure*' OR worker*):ti)	#4	TI=("occupational exposure*" OR worker*)
#5	#3 NOT #4	#5	#3 NOT #4	#5	#3 NOT #4
#6	Neoplasms[mh] OR neoplasm*[tiab] OR cancer*[tiab] OR tumor[tiab] OR tumors[tiab] OR tumour*[tiab] OR neoplasia*[tiab] OR carcinom*[tiab] OR adenocarcinom*[tiab] OR (adenom*[tiab] AND malignan*[tiab]) OR sarcoma[tiab] OR sarcomas[tiab] OR adenosarcoma*[tiab] OR carcinosarcoma*[tiab]	#6	neoplasm/exp OR (neoplasm* OR cancer* OR tumor OR tumors OR tumour* OR neoplasia* OR carcinom* OR adenocarci- nom* OR (adenom* AND malignan*) OR sarcoma OR sarcomas OR adenosarcoma* OR carcinosarcoma*):ti,ab	#6	TS=(neoplasm* OR cancer* OR tumor OR tumors OR tumour* OR neoplasia* OR carcinom* OR adenocarcinom* OR (adenom* AND malignan*) OR sarcoma OR sarcomas OR adenosarcoma* OR carcinosarcoma*)
#7	#5 AND #6	#7	#5 AND #6	#7	#5 AND #6
#8	Risk Factors[mh:noexp] OR Risk[mh:noexp] OR Incidence[mh] OR Mortality[mh:noexp] OR risk[tiab] OR risks[tiab] OR incidence[tiab] OR mortality[tiab] OR death rate*[tiab] OR etiology[sh] OR etiology[tiab] OR cause[tiab] OR causes[tiab] OR caused[tiab] OR causing[tiab] OR causative[tiab] OR causality[tiab] OR due to[tiab] OR epidemiol*[tiab] OR hospitalization[tiab] OR hospitalisation[tiab] OR in hospital[tiab]	#8	risk/de OR 'risk factor'/de OR incidence/ de OR 'cancer incidence'/de OR mortality/de OR 'cancer mortality'/de OR etiology/lnk OR (risk OR risks OR incidence OR mortality OR 'death rate*' OR etiology OR cause OR causes OR caused OR causing OR causative OR causality OR 'due to' OR epidemiol* OR hospitali*ation OR 'in hospital'):ti,ab	#8	TS=(risk OR risks OR incidence OR mor- tality OR "death rate*" OR etiology OR cause OR causes OR caused OR causing OR causative OR causality OR "due to" OR epidemiol* OR hospitali*ation OR "in hospital")
#9	#7 AND #8	#9	#7 AND #8	#9	TS=("case control" OR cohort OR follow-up OR longitudinal OR retro- spective OR prospective)
#10	(Animals[mh] NOT Humans[mh]) OR Models, Animal[mh:noexp] OR Disease Models, Animal[mh] OR Animal Experimentation[mh]	#10	(animal/exp NOT human/exp) OR 'animal model'/exp OR 'animal experiment'/exp OR 'animal cell'/de OR 'animal tissue'/de OR 'in vitro study'/de OR 'nonhuman'/de	#10	#7 AND #8 AND #9
#11	Case-Control Studies[mh] OR Cohort Studies[mh] OR case control[tiab] OR cohort[tiab] OR follow-up[tiab] OR longitudinal[tiab] OR retrospective[tiab] OR prospective[tiab]	#11	('case control study'/exp OR 'cohort analysis'/de OR 'prospective study'/de OR 'retrospective study'/de OR 'longitudinal study'/de OR 'follow up'/de OR ('case control' OR cohort OR follow-up OR longitudinal OR retrospective OR prospective):ti,ab)	#11	English literature
#12	English[la]	#12	[english]/lim		
#13	#9 NOT #10 AND #11 AND #12	#13	#9 NOT #10 AND #11 AND #12		

**Table 2.** Characteristics of the included studies

Study	Country	Study period	Study type	Incinerator type	Exposure	Outcome	Effect size type	Cancer type	Sex	Total samples	Exposure category	Covariates	NOS
Viel et al., 2008	France	1993-1998	Case-control	MWI	Dispersion model (dioxin)	OR	OR	Breast	Female	414 cases, 2,170 controls	<0.0001 pg/m^3^, 0.0001-0.0002 pg/m^3^, 0.0002-0.0004 pg/m^3^, 0.0004-0.0016 pg/m^3^	None (stratified by age)	5
Pronk et al., 2013	USA	1998-2001	Case-control	Medical, municipal, sewage, hazardous waste incinerators	Distance	OR	OR	NHL	Both	1,321 cases, 1,057 controls	Residential proximity to incinerator (3, 5 km) Years (0, 1-14, ≥15)	Age, sex, race, study center, education	7
Floret et al., 2003	France	1980-1996	Case-control	MWI	Dispersion model (dioxin)	OR	OR	NHL	Both	2,442 cases, 2,220 controls	<0.0001 pg/m^3^, 0.0001-0.0002 pg/m^3^, 0.0002-0.0004 pg/m^3^, 0.0004-0.0016 pg/m^3^	None (sex, age and residence block were considered in control selection)	6
Comba et al., 2003	Italy	1989-1999	Case-control	Industrial waste incinerator	Distance	OR	OR	Sarcoma	Both	37 cases, 171 controls	Distance from incinerator (<2, 2-3, 3-4, 4-5, >5 km)	Age, sex	7
Zambon et al., 2007	Italy	1990-1996	Case-control	Industrial waste incinerators, municipal solid incinerators, medical waste incinerators, thermal power plants, oil refineries, INDs for the production of primary aluminum	Dispersion model (PCDD/PCDF)	OR	OR	Sarcoma	Both	44 cases, 93 controls	Exposure: <4 fgr/m^3^, 4-6 fgr/m^3^, ≥6 fgr/m^3^, length: <32, ≥32 yr	None (sex and age were considered in control selection)	7
Bendetti et al., 2020	Italy	1999-2015	Case-control	Industrial waste incinerator	Region	OR	OR	Sarcoma	Both	391 cases, 1,564 controls	Frassino, Virgiliana, Lunetta, Valetta- Valsecchi region (chosen by dispersion model based on SO_2_)	Occupational history (sex and age were considered in control selection)	8
Biggeri et al., 1996	Italy	1979-1981, 1985-1987	Case-control	Incinerator (unspecified)	Distance	OR (with death cases)	RR	Lung	Male	755 cases, 755 controls	Distance from pollutant sources	Age, smoking, occupational exposure, levels of air particulate and excess risk as function of distance from the city center	8
Ranzi et al., 2011	Italy	1990-2003	Cohort	MWI	Dispersion model (heavy metal)	Incidence, mortality	RR	Stomach, colorectal, liver, larynx, lung, sarcoma, prostate, bladder, CNS, lympho-hematopoietic, NHL, myeloma, leukemia	Both (separately)	31,347 (354,702 person-yr)	Quartiles, <0.5 ng/m^3^, 0.5-1 ng/m^3^, 1-2 ng/m^3^, >2 ng/m^3^	Age, socioeconomic status	7
VoPham et al., 2020	USA	1989-2013	Cohort	MWI	Distance	Incidence	HR	Breast cancer	Female	112,397 (2,302,566 person-yr)	Residential proximity to incinerator (3, 5, 10 km) and length (0, 1-6, ≥6 yr)	Age, race, family history of breast cancer, personal history of biopsy-confirmed BBD, age at menarche, parity, age at first birth, lactation, menopausal status and hormone use, height, BMI at age 18, change in BMI since age 18. physical activity, smoking status, adult alcohol consumption, census tract median home value, census tract median income, marital status, living arrangements, individual-level income, population density	6
Ancona et al., 2015	Italy	2001-2011	Cohort	MWI	Dispersion model (PM_10_)	Hospital admission, mortality	HR	Stomach, colorectal, liver, pancreas, larynx, lung, breast, bladder, kidney, brain, lympho-hematopoietic	Both (separately)	85,559 (720,452 person-yr)	5th vs. 95th (equal to linear increase for 0.027 n/m^3^ of PM_10_)	Age, education, occupation, civil status, area-based SEP index, outdoor NO_2_ concentration	7
Romanelli et al., 2019	Italy	2001-2014	Cohort	MWI	Dispersion model (NOx-MWI)	Hospitalization, mortality	HR	Stomach, colorectal, liver, lung, connective tissue, lympho-hematopoietic, CNS, leukemia, NHL, lung	Both (separately)	132,293 (1,092,817 person-yr)	NOx-MWI – <0.013 μg/m^3^, 0.013-0.0194 μg/m^3^, 0.0194-0.0301 μg/m^3^, 0.0301-0.558 μg/m^3^	Age, other environmental sources (NOx-IND, NOx-TR), and deprivation index	7

NOS, Newcastle-Ottawa Scale; MWI, municipal waste incinerator; OR, odds ratio; NHL, non-Hodgkin lymphoma; PCDD, polychlorinated dibenzo-p-dioxin; PCDF, polychlorinated dibenzofuran; SO_2_, sulfur dioxide; RR, risk ratio; HR, hazard ratio; CNS, central nervous system; BBD, benign breast disease; BMI, body mass index; PM_10_, particulate matter less than 10 μm; NO_2_, nitrogen dioxide; SEP, socioeconomic position; NOx, nitric oxide (NO) and nitrogen dioxide (NO_2_); IND, industrial plant; TR, traffic-related.

## SUPPLEMENTARY MATERIALS

Supplementary Material 1.

Supplementary Material 2. Quality assessment of included literature by the Newcastle-Ottawa Scale

Supplementary Material 3.

Supplementary Material 4. Funnel plot and Egger’s test for assessing publication bias by cancer type

Supplementary Material 5. Pooled risk estimates by subgroup analysis
