# Supplementary material for: Systematic review and meta-analysis of cancer risks in relation to environmental waste incinerator emissions: a meta-analysis of case-control and cohort studies
Source: Epidemiol Health. 2022 Sep 1;44:e2022070. doi: 10.4178/epih.e2022070 (PMC9849852; doi:10.4178/epih.e2022070)
Supplement: Supplementary Material 1. [file epih-44-e2022070-suppl1.docx]

| Author | Year | Title | Inclusion | Reason of exclusion |
| --- | --- | --- | --- | --- |
| A. Biggeri et al. | 1996 | Air pollution and lung cancer in Trieste, Italy: spatial analysis of risk as a function of distance from sources | Included | - |
| N. Floret et al. | 2003 | Dioxin emissions from a solid waste incinerator and risk of non-Hodgkin lymphoma | Included | - |
| P. Zambon et al. | 2007 | Sarcoma risk and dioxin emissions from incinerators and industrial plants: a population-based case-control study (Italy) | Included | - |
| J. F. Viel et al. | 2008 | Dioxin emissions from a municipal solid waste incinerator and risk of invasive breast cancer: a population-based case-control study with GIS-derived exposure | Included | - |
| A. Ranzi et al. | 2011 | Mortality and morbidity among people living close to incinerators: a cohort study based on dispersion modeling for exposure assessment | Included | - |
| A. Pronk et al. | 2013 | Residential proximity to industrial combustion facilities and risk of non-Hodgkin lymphoma: a case-control study | Included | - |
| C. Ancona et al. | 2015 | Mortality and morbidity in a population exposed to multiple sources of air pollution: A retrospective cohort study using air dispersion models | Included | - |
| A. M. Romanelli et al. | 2019 | Mortality and Morbidity in a Population Exposed to Emission from a Municipal Waste Incinerator. A Retrospective Cohort Study | Included | - |
| T. VoPham et al. | 2020 | Dioxin exposure and breast cancer risk in a prospective cohort study | Included | - |
| M. Benedetti et al. | 2020 | Residential proximity to an industrial incinerator and risk of soft-tissue sarcoma, 1999-2014 | Included | - |
| P. Comba et al. | 2003 | Risk of soft tissue sarcomas and residence in the neighbourhood of an incinerator of industrial wastes | Included | - |
| S. W. Hu et al. | 2001 | Waste incineration and pulmonary function: An epidemiologic study of six communities | Excluded | No information on cancer risk |
| F. Forastiere et al. | 2011 | Health impact assessment of waste management facilities in three European countries | Excluded | No information on cancer risk |
| M. Saintot et al. | 2004 | Interaction between genetic polymorphism of cytochrome P450-1B1 and environmental pollutants in breast cancer risk | Excluded | Study on specific population |
| P. A. Bertazzi et al. | 1989 | Lung cancer in relation to residence in census tracts with toxic-waste disposal sites: a case-control study in Niagara County, New York | Excluded | No information on incinerator emission exposure |
| P. A. Bertazzi et al. | 1993 | CANCER INCIDENCE IN A POPULATION ACCIDENTALLY EXPOSED TO 2,3,7,8-TETRACHLORODIBENZO-PARA-DIOXIN | Excluded | No information on incinerator emission exposure |
| F. Barbone et al. | 1995 | AIR-POLLUTION AND LUNG-CANCER IN TRIESTE, ITALY | Excluded | No information on incinerator emission exposure |
| L. Hardell et al. | 1996 | Increased concentrations of octachlorodibenzo-p-dioxin in cases with breast cancer - Results from a case-control study | Excluded | No information on incinerator emission exposure |
| B. Revich et al. | 2001 | Dioxin exposure and public health in Chapaevsk, Russia | Excluded | No information on incinerator emission exposure |
| P. Nafstad et al. | 2003 | Lung cancer and air pollution: a 27 year follow up of 16 209 Norwegian men | Excluded | No information on incinerator emission exposure |
| J. T. Tuomisto et al. | 2004 | Soft-tissue sarcoma and dioxin: A case-control study | Excluded | No information on incinerator emission exposure |
| P. Vineis et al. | 2007 | Lung cancers attributable to environmental tobacco smoke and air pollution in non-smokers in different European countries: a prospective study | Excluded | No information on incinerator emission exposure |
| J. Czarnota et al. | 2015 | Analysis of Environmental Chemical Mixtures and Non-Hodgkin Lymphoma Risk in the NCI-SEER NHL Study | Excluded | No information on incinerator emission exposure |
| A. Danjou et al. | 2015 | Environmental dioxin exposure and risk of breast cancer: The geo3n pilot study in the rhône-alpes region, France | Excluded | No information on incinerator emission exposure |
| F. Mataloni et al. | 2016 | Morbidity and mortality of people who live close to municipal waste landfills: a multisite cohort study | Excluded | No information on incinerator emission exposure |
| N. Borciani et al. | 2011 | Municipal solid waste incineration and risk of soft-tissue sarcoma: A case-control study | Excluded | Conference abstract |
| R. R. Jones et al. | 2021 | Residential proximity to dioxinemitting facilities and risk of non-hodgkin lymphoma in the NIH-AARP diet and health study | Excluded | Conference abstract |
| P. A. Bertazzi et al. | 2001 | Health effects of dioxin exposure: A 20-year mortality study | Excluded | Review article |
| P. Diggle et al. | 1999 | Case-control isotonic regression for investigation of elevation in risk around a point source | Excluded | Study on methodology |
| F. Minichilli et al. | 2016 | Epidemiological population-based cohort study on mortality and hospitalization in the area near the waste incinerator plant of San Zeno, Arezzo (Tuscany Region, Central Italy) | Excluded | Not in English |
| E. Chellini et al. | 2020 | Epidemiological study on the population resident in the neighbourhood of an incinerator in Tuscany Region (Central Italy) | Excluded | Not in English |
| N. Floret et al. | 2007 | A municipal solid waste incinerator as the single dominant point source of PCDD/Fs in an area of increased non-Hodgkin's lymphoma incidence | Excluded | Study design is neither cohort nor case-control |
| P. Elliott et al. | 1992 | INCIDENCE OF CANCERS OF THE LARYNX AND LUNG NEAR INCINERATORS OF WASTE SOLVENTS AND OILS IN GREAT-BRITAIN | Excluded | Study design is neither cohort nor case-control |
| P. Elliott et al. | 1996 | Cancer incidence near municipal solid waste incinerators in Great Britain | Excluded | Study design is neither cohort nor case-control |
| R. C. Pleus and K. E. Kelly | 1996 | Health effects from hazardous waste incineration facilities: five case studies | Excluded | Study design is neither cohort nor case-control |
| J. F. Viel et al. | 2000 | Soft-tissue sarcoma and non-Hodgkin's lymphoma clusters around a municipal solid waste incinerator with high dioxin emission levels | Excluded | Study design is neither cohort nor case-control |
| J. Garcia-Perez et al. | 2009 | Mortality due to lung, laryngeal and bladder cancer in towns lying in the vicinity of combustion installations | Excluded | Study design is neither cohort nor case-control |
| M. Federico et al. | 2010 | Cancer incidence in people with residential exposure to a municipal waste incinerator: an ecological study in Modena (Italy), 1991-2005 | Excluded | Study design is neither cohort nor case-control |
| C. Salerno et al. | 2015 | Exploration study on mortality trends in the territory surrounding an incineration plant of urban solid waste in the municipality of Vercelli (Piedmont, Italy) 1988-2009 | Excluded | Study design is neither cohort nor case-control |
| C. Salerno et al. | 2017 | Geographical and epidemiological analysis of oncological incidence in paediatric and adolescent ages in a municipality of North-Western Italy: Vercelli, years 2002-2009 | Excluded | Study design is neither cohort nor case-control |
| E. M. Barjoan et al. | 2020 | Cancer incidence in the vicinity of a waste incineration plant in the Nice area between 2005 and 2014 | Excluded | Study design is neither cohort nor case-control |
| C. F. Samer et al. | 2020 | Cytochrome P450 1A2 activity and incidence of thyroid disease and cancer after chronic or acute exposure to dioxins | Excluded | Study design is neither cohort nor case-control |
