# Supplementary material for: Systematic review and meta-analysis of cancer risks in relation to environmental waste incinerator emissions: a meta-analysis of case-control and cohort studies
Source: Epidemiol Health. 2022 Sep 1;44:e2022070. doi: 10.4178/epih.e2022070 (PMC9849852; doi:10.4178/epih.e2022070)
Supplement: Supplementary Material 2. — Quality assessment of included literature by the Newcastle-Ottawa Scale [file epih-44-e2022070-suppl2.docx]

| Table S2. Quality assessment of included literature by the Newcastle-Ottawa Scale | | | | | |  |  |  |  |  |
| --- | --- | --- | --- | --- | --- | --- | --- | --- | --- | --- |
|  |  | Selection | | | | Comparability | Exposure | | |  |
|  |  | 1 | 2 | 3 | 4 | 1 | 1 | 2 | 3 | Score |
| Case-control study | Viel JF, 2008 |  | ***** | ***** |  | ***** | ***** | ***** |  | 5 |
|  | Pronk A, 2013 | ***** | ***** | ***** |  | ****** | ***** | ***** |  | 7 |
|  | Floret N, 2003 | ***** | ***** | ***** | ***** |  | ***** | ***** |  | 6 |
|  | P Comba, 2003 | ***** | ***** | ***** |  | ****** | ***** | ***** |  | 7 |
|  | Zambon P, 2007 | ***** | ***** | ***** | ***** | ***** | ***** | ***** |  | 7 |
|  | Bendetti, 2020 | ***** | ***** | ***** | ***** | ****** | ***** | ***** |  | 8 |
|  | Biggeri, 1996 | ***** | ***** | ***** | ***** | ****** | ***** | ***** |  | 8 |
|  |  | Selection | | | | Comparability | Outcome | | |  |
|  |  | 1 | 2 | 3 | 4 | 1 | 1 | 2 | 3 | Score |
| Cohort study | Ranzi, 2011) | ***** | ***** | ***** |  | ****** | ***** | ***** |  | 7 |
|  | VoPham T, 2020 |  | ***** | ***** |  | ****** | ***** | ***** |  | 6 |
|  | Ancona, 2015 | ***** | ***** | ***** |  | ****** | ***** | ***** |  | 7 |
|  | Romanelli, 2019 | ***** | ***** | ***** |  | ****** | ***** | ***** |  | 7 |
|  |  |  |  |  |  |  |  |  |  |  |
