# Supplementary figures and images for: Systematic review and meta-analysis of cancer risks in relation to environmental waste incinerator emissions: a meta-analysis of case-control and cohort studies
Source: Epidemiol Health. 2022 Sep 1;44:e2022070. doi: 10.4178/epih.e2022070 (PMC9849852; doi:10.4178/epih.e2022070)

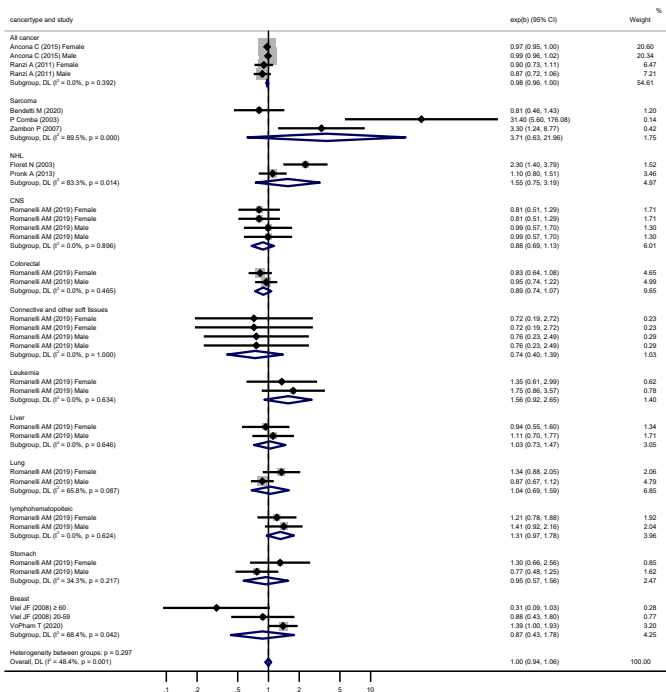

NOTE: Weights and between-subgroup heterogeneity test are from random-effects model

Supplement: Supplementary Material 3. [file epih-44-e2022070-suppl3.pdf]
