# Supplementary material for: Systematic review and meta-analysis of cancer risks in relation to environmental waste incinerator emissions: a meta-analysis of case-control and cohort studies
Source: Epidemiol Health. 2022 Sep 1;44:e2022070. doi: 10.4178/epih.e2022070 (PMC9849852; doi:10.4178/epih.e2022070)

Supplement 4. Funnel plot and Egger’s test for assessing publication bias by cancer type

Breast cancer

Egger’s test: Coefficient (95% CI): 0.34(-0.11, 0.78) p = 0.07

Funnel Plot


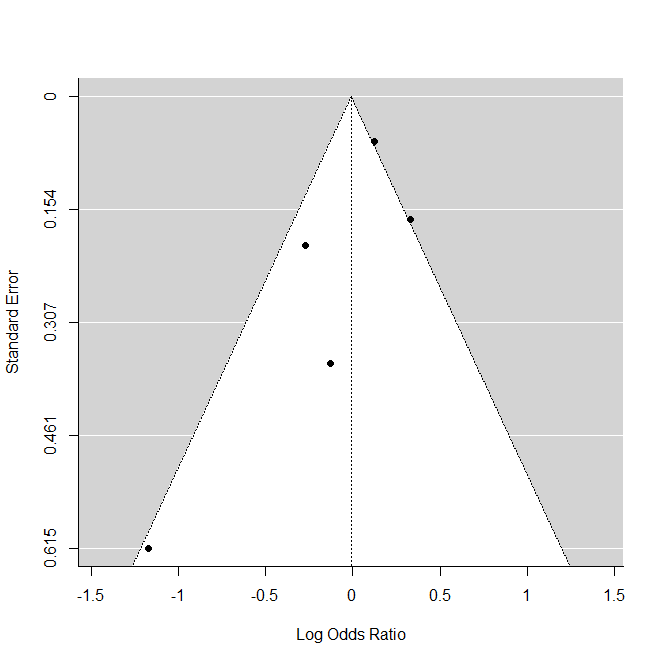


Colorectal cancer

Egger’s test: Coefficient (95% CI): -0.29 (-0.64, 0.06) p = 0.27

Funnel plot


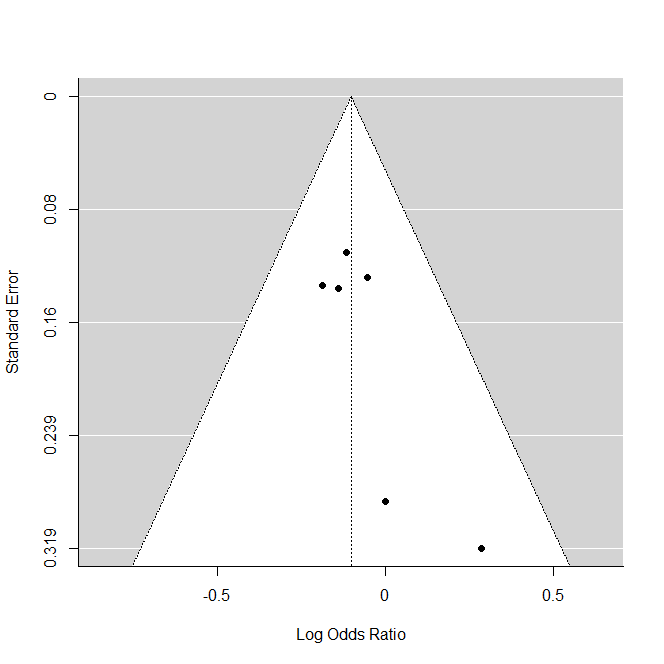


Liver cancer

Egger’s test: Coefficient (95% CI): 0.49 (-0.07, 1.04) p=0.12

Funnel plot


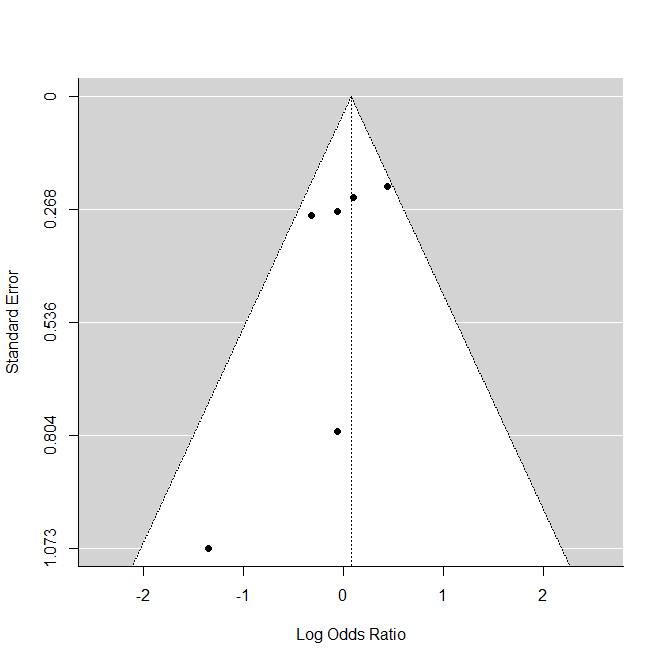


Lung cancer

Egger’s test: Coefficient (95% CI): -0.01 (-0.26, 0.23) p = 0.96

Funnel Plot


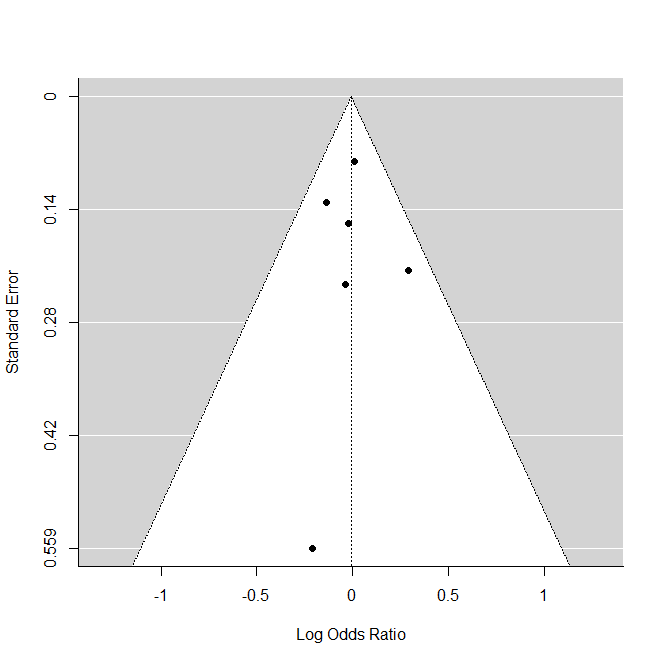


Lymphohematopoiteic cancer

Egger’s test: Coefficient (95% CI): -0.16 (-0.56, 0.24) p = 0.56

Funnel Plot


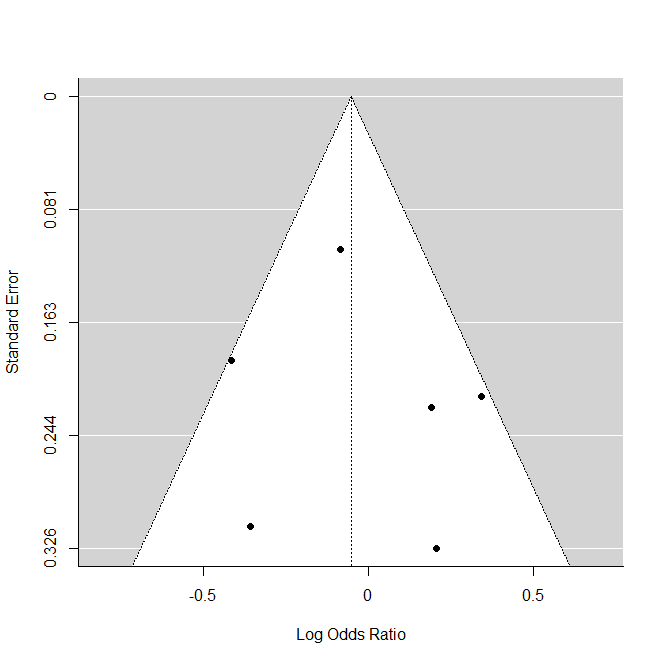


NHL

Egger’s test: Coefficient (95% CI): 0.99 (-1.24, 3.22) p=0.31

Funnel Plot


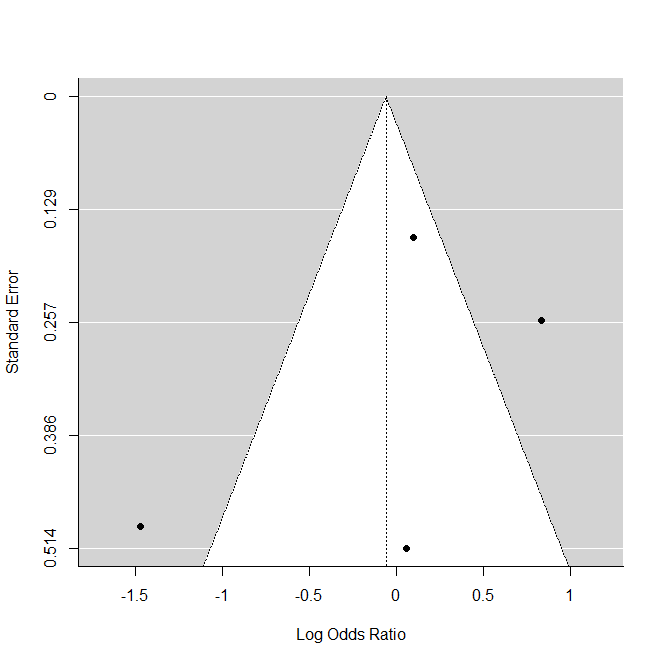


Sarcoma

Egger’s test: Coefficient (95% CI): -0.24 (-3.97, 4.45) p = 0.68

Funnel Plot


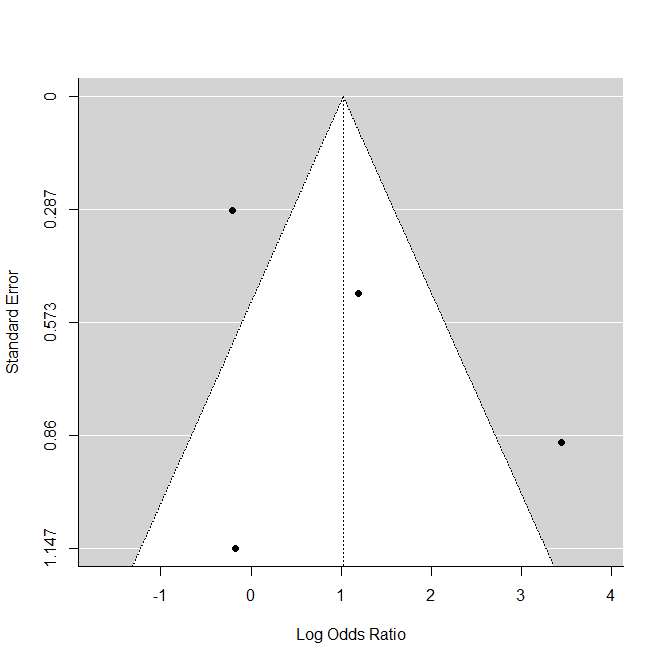


Stomach cancer

Egger’s test: Coefficient (95% CI): -0.25 (-0.90, 0.39) p=0.46

Funnel Plot


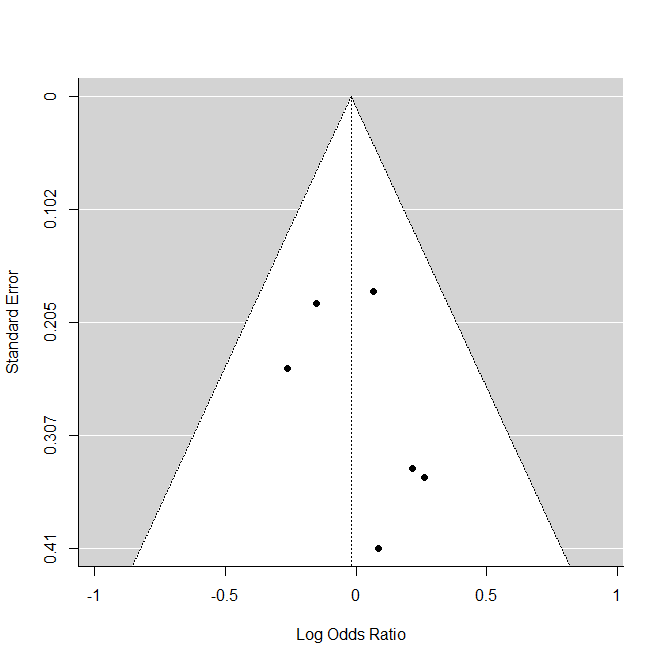


All cancer (from literature reporting ‘all cancer’)

Egger’s test: Coefficient (95% CI): -0.01 (-0.04, 0.03) p=0.19

Funnel Plot
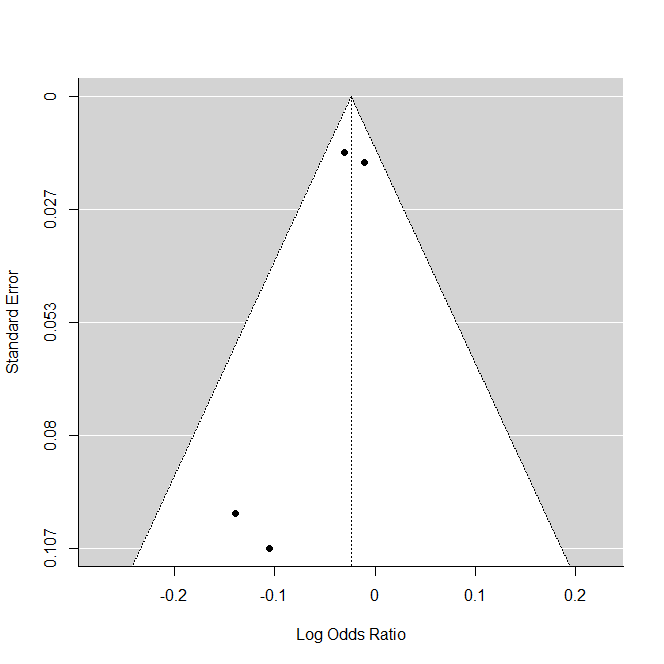


Bladder cancer

Egger’s test: Coefficient (95% CI): -0.22 (-0.95, 0.51) p=0.55

Funnel Plot


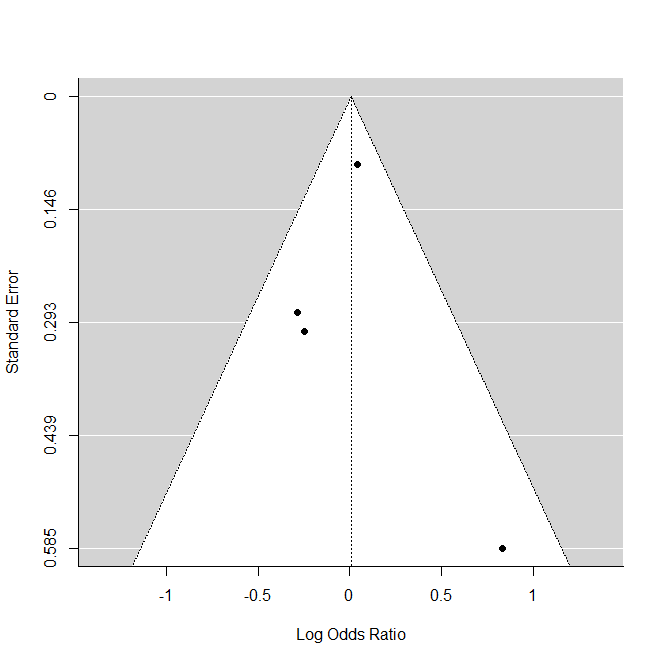


CNS cancer

Egger’s test: Coefficient (95% CI): -0.40 (-1.35, 0.55) p = 0.50

Funnel Plot


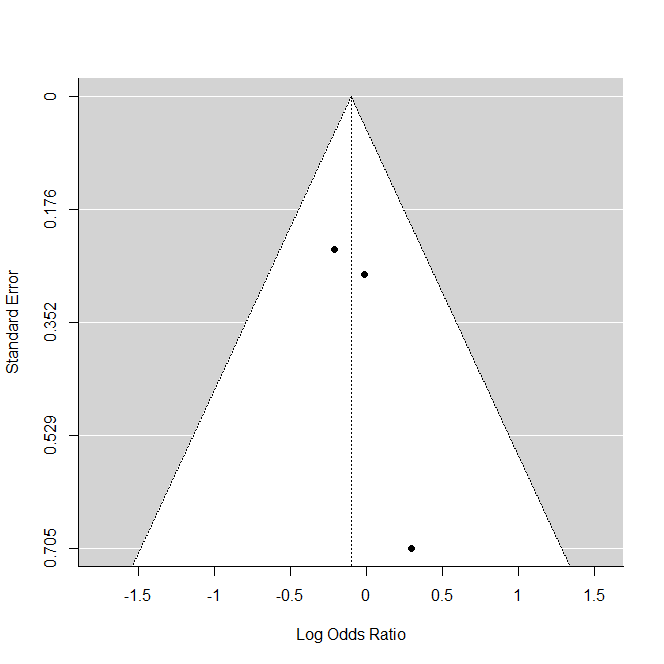


Laryngeal cancer

Egger’s test: Coefficient (95% CI): 0.58 (-0.29, 1.45) p = 0.25

Funnel Plot


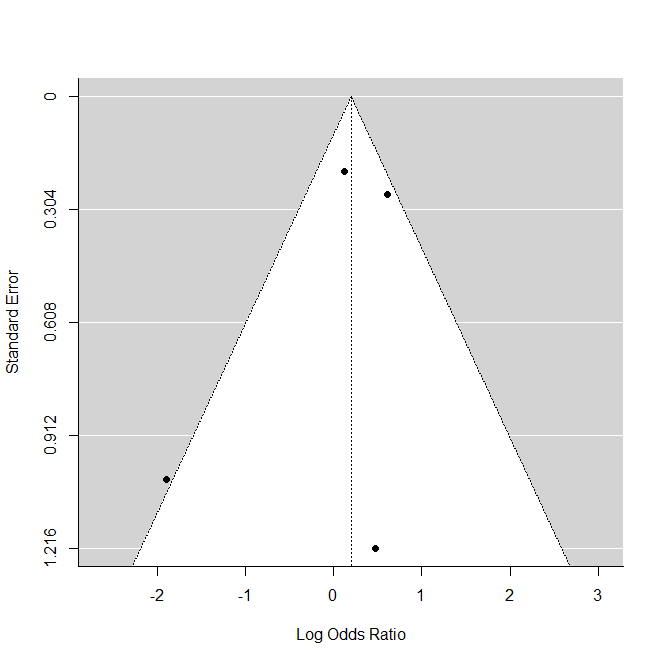


Leukemia

Egger’s test: Coefficient (95% CI): 0.99 (-1.01, 2.98) p = 0.51

Funnel Plot


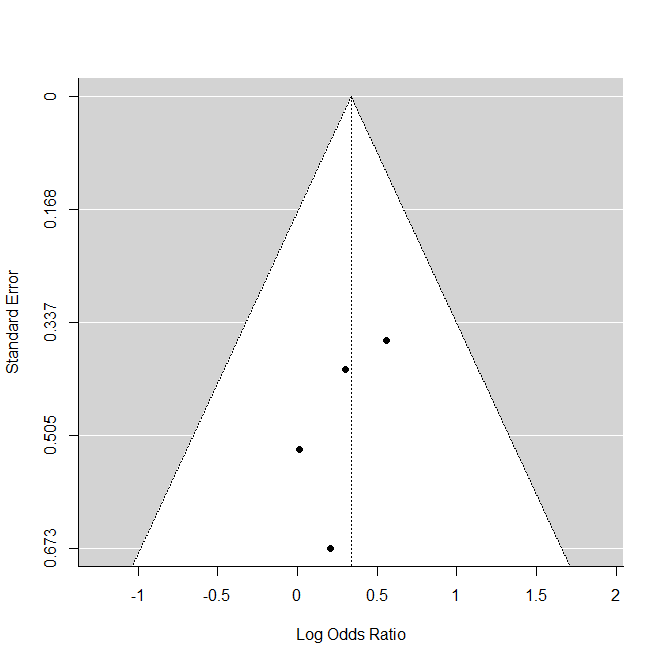


All cancer (pooled all effect size identified regardless of types)

Egger’s test: Coefficient (95% CI): -0.03 (-0.05, -0.01) p = 0.08

Funnel Plot


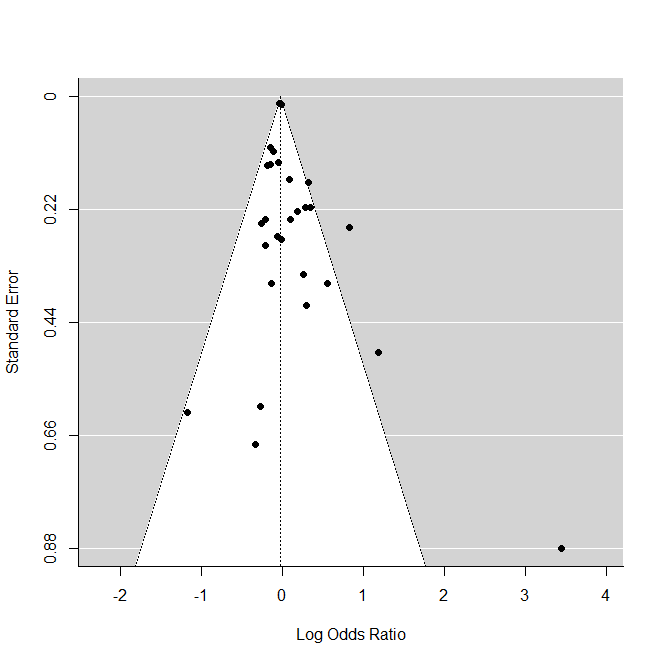

Supplement: Supplementary Material 4. — Funnel plot and Egger’s test for assessing publication bias by cancer type [file epih-44-e2022070-suppl4.docx]
