# Supplementary material for: Systematic review and meta-analysis of cancer risks in relation to environmental waste incinerator emissions: a meta-analysis of case-control and cohort studies
Source: Epidemiol Health. 2022 Sep 1;44:e2022070. doi: 10.4178/epih.e2022070 (PMC9849852; doi:10.4178/epih.e2022070)
Supplement: Supplementary Material 5. — Pooled risk estimates by subgroup analysis [file epih-44-e2022070-suppl5.docx]

| Table S5. Pooled risk estimates by subgroup analysis | | | |  |  |
| --- | --- | --- | --- | --- | --- |
|  | Number of studies | Number of effect size | Pooled effect size (95% CI) | Heterogeneity | |
|  |  |  |  | I^2^ statistics (%) | P value (Cochran's Q test) |
| By sex |  |  |  |  |  |
| Male |  |  |  |  |  |
| All cancer | 2 | 2 | 0.96 (0.87, 1.07) | 40.3 | 0.20 |
| Bladder | 2 | 2 | 1.02 (0.86, 1.2) | 0.0 | 0.94 |
| CNS | 2 | 2 | 1.03 (0.62, 1.71) | 0.0 | 0.68 |
| Colorectal | 3 | 3 | 0.92 (0.79, 1.08) | 0.0 | 0.89 |
| Larynx | 2 | 2 | 0.53 (0.08, 3.6) | 72.9 | 0.06 |
| Leukemia | 2 | 2 | 1.47 (0.82, 2.63) | 0.0 | 0.39 |
| Liver | 3 | 3 | 0.87 (0.56, 1.36) | 24.8 | 0.26 |
| Lung | 3 | 3 | 0.97 (0.85, 1.10) | 0.0 | 0.63 |
| Lymphohematopoietic | 3 | 3 | 0.99 (0.71, 1.39) | 53.7 | 0.12 |
| NHL | 2 | 2 | 0.67 (0.09, 5.15) | 91.2 | 0.00 |
| Stomach | 3 | 3 | 0.88 (0.68, 1.15) | 0.0 | 0.51 |
| Female |  |  |  |  |  |
| All cancer | 2 | 2 | 0.97 (0.94, 0.99) | 0.0 | 0.49 |
| Bladder | 2 | 2 | 1.17 (0.40, 3.42) | 66.5 | 0.08 |
| Breast | 4 | 5 | 1 (0.76, 1.32) | 60.3 | 0.04 |
| Colorectal | 3 | 3 | 0.88 (0.74, 1.05) | 0.0 | 0.39 |
| Larynx | 2 | 2 | 1.82 (1.10, 3.01) | 0.0 | 0.91 |
| Leukemia | 2 | 2 | 1.32 (0.67, 2.6) | 0.0 | 0.91 |
| Liver | 3 | 3 | 1.25 (0.88, 1.78) | 10.6 | 0.33 |
| Lung | 3 | 3 | 1.08 (0.84, 1.37) | 0.0 | 0.44 |
| Lymphohematopoietic | 3 | 3 | 0.96 (0.62, 1.49) | 62.3 | 0.07 |
| NHL | 2 | 2 | 1.36 (0.76, 2.43) | 0.0 | 0.55 |
| Stomach | 3 | 3 | 1.11 (0.83, 1.48) | 0.0 | 0.88 |
| By exposure estimation method | |  |  |  |  |
| Modeling |  |  |  |  |  |
| All cancer | 2 | 4 | 0.98 (0.96, 1.00) | 0.0 | 0.39 |
| Bladder | 2 | 4 | 0.98 (0.75, 1.26) | 23.7 | 0.27 |
| Breast | 3 | 4 | 0.86 (0.59, 1.26) | 62.4 | 0.05 |
| CNS | 2 | 3 | 0.91 (0.64, 1.28) | 0.0 | 0.73 |
| Colorectal | 3 | 6 | 0.90 (0.80, 1.02) | 0.0 | 0.81 |
| Larynx | 2 | 4 | 1.19 (0.65, 2.21) | 54.4 | 0.09 |
| Leukemia | 2 | 4 | 1.40 (0.90, 2.19) | 0.0 | 0.85 |
| Liver | 3 | 6 | 1.05 (0.78, 1.41) | 27.2 | 0.23 |
| Lung | 3 | 6 | 0.99 (0.89, 1.11) | 0.0 | 0.68 |
| Lymphohematopoietic | 3 | 6 | 0.97 (0.77, 1.23) | 49.1 | 0.08 |
| NHL | 3 | 5 | 1.17 (0.57, 2.4) | 78.0 | 0.00 |
| Sarcoma | 2 | 2 | 2.46 (0.82, 7.4) | 16.5 | 0.27 |
| Stomach | 3 | 6 | 0.98 (0.81, 1.19) | 0.0 | 0.71 |
| By outcome type |  |  |  |  |  |
| Mortality |  |  |  |  |  |
| All cancer | 2 | 4 | 1.12 (0.95, 1.32) | 72.8 | 0.01 |
| Bladder | 2 | 4 | 1.22 (0.88, 1.7) | 0.0 | 0.51 |
| Breast | 2 | 2 | 1.30 (0.65, 2.61) | 71.2 | 0.06 |
| Colorectal | 3 | 6 | 1.06 (0.83, 1.34) | 19.3 | 0.29 |
| Leukemia | 2 | 4 | 1.64 (0.73, 3.7) | 0.0 | 0.50 |
| Liver | 3 | 6 | 1.04 (0.69, 1.56) | 26.9 | 0.23 |
| Lung | 4 | 7 | 1.07 (0.92, 1.23) | 72.8 | 0.19 |
| Lymphohematopoietic | 3 | 6 | 1.10 (0.77, 1.57) | 52.4 | 0.06 |
| NHL | 2 | 4 | 1.49 (0.84, 2.66) | 0.0 | 0.45 |
| Stomach | 3 | 6 | 0.96 (0.76, 1.23) | 0.0 | 0.77 |
|  |  |  |  |  |  |
